# Identifying eating habits in Finnish children: a cross-sectional study

**DOI:** 10.1186/s12889-019-6603-x

**Published:** 2019-03-15

**Authors:** Rejane Augusta de Oliveira Figueiredo, Jannina Viljakainen, Heli Viljakainen, Eva Roos, Trine B. Rounge, Elisabete Weiderpass

**Affiliations:** 1 Folkhälsan Research Center, Biomedicum 1 Helsinki, PB 63 (Haartmansgatan 8), 00014 University of Helsinki, Helsinki, Finland; 2 Faculty of Medicine, University of Helsinki, Helsinki, Finland; 3 Department of Food and Environmental Sciences, University of Helsinki, Helsinki, Finland; 4 Department of Public Health, University of Helsinki, Helsinki, Finland; 5 Department of Research, Cancer Registry of Norway, Oslo, Norway; 6 Department of Medical Epidemiology and Biostatistics, Karolinska Institutet, Stockholm, Sweden; 7 Department of Community Medicine, Faculty of Health Sciences, University of Tromsø, The Arctic University of Norway, Tromsø, Norway

**Keywords:** Eating habits, Healthy eating, Breakfast, Meal pattern, Children, Finland, Epidemiology

## Abstract

****Background**:**

We aimed to identify different eating habits among Finnish children and to evaluate their association with meal patterns, breakfast consumption, and socio-demographic characteristics in a large, nationwide cohort of children.

****Methods**:**

We evaluated 10,569 children aged 9–14 years into the Finnish Health in Teens cohort in a cross-sectional design. The hierarchical K-means method was used to identify groups of children with different eating habits, based on five factors obtained through factor analysis of 10 food items. Multiple correspondence analysis was used to show associations between groups with different eating habits and meal patterns, breakfast patterns, gender, age, and language spoken at home.

****Results**:**

Analyses identified three groups: unhealthy eaters (12.3%), fruit and vegetable avoiders (43.3%), and healthy eaters (44.1%). Most children had regular meal and breakfast patterns. The proportion of boys was higher among unhealthy eaters. Unhealthy eaters also showed irregular meal and breakfast patterns, and had parents with low education level. There was a higher proportion of girls among healthy eaters. Healthy eaters also showed regular meal and breakfast patterns, and had parents with high education level.

****Conclusions**:**

Although the number of unhealthy eaters was small, special attention should be still paid to these, mostly male children, as they have poor eating habits and they lack regular eating routine. Skipping breakfast was more common among older children and girls, although girls had healthier eating habits overall. Our results can contribute to public health efforts to improve eating behaviours, especially among children with poor eating habits and those skipping healthy food items.

## Background

At present, countries worldwide are focusing on fostering a healthy diet and healthy eating habits, which are major determinants of health and disease [1], including the development of overweight and obesity [2]. Due to the pandemic of childhood obesity [3], the eating habits of children and adolescents are of particular importance, as unhealthy eating habits in childhood/adolescence can persist and cause adverse health outcomes in adulthood [4, 5].

A study carried out in 124 developed and developing countries showed an improvement in worldwide dietary quality from 1980 to 2009, with an increased availability of energy from vegetable oils, fruits, and vegetables, and a decreased availability of energy from sugar and animal fats [6]. Over the last years, the Finnish diet among working-age adults has also improved, with an increase in the consumption of fruits and vegetables and a decrease in the consumption of sweets and soft drinks [7, 8].

However, Hoppu et al. [9] reported that the main dietary concerns among Finnish adolescents are low consumption of fruits and vegetables and high consumption of sucrose-rich drinks and snacks. Other studies have reported that young children commonly consume skimmed milk, low-fat cheese or cold-cuts, and vegetable oil-based margarine on bread, but rarely fish [10, 11]. It was estimated that beverages and foods consumed between meals provide as much as 42% of total daily energy intake, and the quality of these food items is of concern [11]. Similar results have been reported in young male conscripts in Finland [12], among whom daily consumption of fruits, berries, and vegetables was rare, and consumption of rye bread, dairy products, and sugar-sweetened soft drinks was favoured. On average, the food consumption of these young men fulfilled less than half of the Nordic Nutrition Recommendations [12].

Healthy eating also includes a consistent meal pattern, and such patterns have been the focus of several studies [13, 14]. The conventional daily Finnish meal pattern includes breakfast, a warm lunch, a warm dinner, and two snacks [15]. Breakfast is consumed daily by 61% of adolescents in Finland, a number which has remained stable between 2002 and 2010 [16], and by 87% of primary school pupils [13]. Among primary and secondary school pupils, 89 and 71%, respectively, have daily school lunch, which is free of charge in public schools in Finland [9, 13].

As it is challenging to distinguish the effect of individual nutrients and foods on health and disease, a whole-diet approach, i.e., describing the combinations in which foods are consumed, is warranted to understand the synergistic and cumulative effects of diet on multiple health outcomes [17]. Few studies have described eating habits in the Finnish young population, and some have evaluated only the intake of specific nutrients and foods in pre-adolescents and adolescents. However, less is known about general eating habits and meal patterns in adolescence [9, 18, 19] and the association of these factors with socio-demographic attributes. Thus we aimed to identify different eating habits among Finnish children and to evaluate the association between these eating habits and meal patterns, breakfast consumption, and socio-demographic characteristics in a large, nationwide cohort of children.

## Methods

### Participants and cohort details

We used data from the Finnish Health in Teens (Fin-HIT) study, a prospective cohort consisting of 11,407 pre-adolescents and adolescents (henceforth denoted as children in this study) and 10,000 parents or other adults responsible for those children (referred as parents in this study), mostly mothers. The participation rate (30%) and details on Fin-HIT cohort were described elsewhere [20]. All children were aged 9–14 years at the time of recruitment, mostly from schools, in 2011–2014. The cohort covered a large part of Finland, including Uusimaa, Varsinais-Suomi, Häme, Pirkanmaa, Keski-Suomi, Pohjois-Savo, and Pohjois-Pohjanmaa.

In the present analysis, we included all children with baseline information on diet, consumption of selected food items, and frequency of consumption of different meals (*n* = 10,569).

### Information on socio-demographic characteristics

Children completed a questionnaire covering various lifestyle and health related topics as previously described [20] . Parents completed a questionnaire which covered information on education level (anything until technical high school was categorized as low education level; anything higher was classified as high education level). Information on gender, age (in years), and language spoken at home (Finnish, Swedish, or other) was obtained from consent forms or questionnaires and confirmed by linkage with the National Population Information System at the Population Register Centre. The language spoken at home was included in the study, which in a way may reflect the participant’s socio-economic status and health conditions. In general, immigrants have the worst health and socio-economic condition [21, 22]. Although Finnish-speakers and the Swedish-speakers have similar living conditions in Finland, studies showed that Swedish-speakers have better socioeconomic status and health condition [23, 24].

### Eating habits and meal information

Information on eating habits in the Fin-HIT study was assessed with a 14-item food frequency questionnaire (FFQ), which covered the preceding month including both school and non-school days. Long questionnaires for children and adolescents can affect their answers, resulting in some bias, so a limited number of food items was included [25, 26]. Selected food items covered the mandatory key indicators to evaluate healthy and unhealthy children’s diet habits, as suggested by the Health Behaviour in School-Aged Children (HBSC) Study protocol [27]. Mandatory items were fruits, vegetables (fresh or cooked), sweets and sugary soft drinks. Other food items included were also important and typically used in European school studies as indicators of healthy (dark grain bread; milk or soured milk; fresh juice; and water) and unhealthy eating behaviors (pizza; hamburger or hot dog; biscuits/cookies; ice cream; chocolate or sweets; salty snacks; sugary juice drinks) [18, 28, 29]. Children self-reported the frequency of consumption of each item on a 7-point scale ranging from 0 (not consumed) to 6 (consumed several times per day).

Information on meal patterns during school days was obtained by the question, “How often do you typically eat following meals during a school week?”, followed by a list of meals: breakfast, school lunch, and dinner. Respondents reported the weekly frequency of consumption of each meal on a 6-point scale ranging from never to 5 days a week. Children who reported consuming lunch and dinner every school day were classified as having a regular consumption on these meals (henceforth denoted as regular meal pattern); all others were classified as having an irregular meal pattern. Breakfast consumption was studied separately since several studies have shown an association between skipping breakfast and adverse health effects [13, 30, 31]. Children who reported consuming breakfast every school day were classified as having a regular breakfast pattern and the others as having an irregular breakfast pattern.

### Ethics

The Fin-HIT study protocol was approved by the Coordinating Ethics Committee of the Helsinki and Uusimaa Hospital District. Informed written consent was obtained from all children and from one legally responsible adult per each child (i.e. parent or legal guardian) according to the Helsinki Declaration.

### Statistical analysis

All associations between categorical variables were assessed using chi-square tests. We then identified groups of children with different eating habits. We had 14 food items, however some items were highly associated, such as cooked vegetables with fresh or grated vegetables, sugary juice with soft drink, fruits or berries with fresh juice, milk with other health items. To avoid overlapping information in the cluster analysis, we excluded food items with strong mutual associations identified by chi-square tests. Therefore, cluster analyses were performed based on 10 food items: pizza; hamburger or hot dog; biscuits/cookies; sweet pastry; ice cream; salty snacks; sugary juice drinks; dark grain bread; fruit or berries; and fresh or grated vegetables or salad. With these 10 items, we carried out a factor analysis using the principal component method for factor extraction and varimax methods for rotation. The applicability of factor analysis model was evaluated by the Kaiser–Meyer–Olklin (KMO) and Bartlett’s sphericity test, considering acceptable values over 0.70 and *p* < 0·05, respectively [32]. To identify groups with different eating habits we used the hierarchical K-means method, using the five factors obtained through factor analysis which represented 70% of the variability of the 10 aforementioned food items. In order to evaluate the robustness of the identified groups, we selected one sample with 60% of the total data and re-ran the cluster analysis. We repeated this process five times and compared the results with those of the original group using the Kappa analysis. All comparisons showed a *p*-value of < 0.001 and a Kappa index greater than 0.7, indicating high agreement.

Multiple correspondence analysis is a descriptive technique which allows researchers to visualize the relationship between several categorical variables in a graphic display [33], the closer the categories, the higher the association between them. In order to visualize different dietary behaviours among children, a multiple correspondence analysis was performed to evaluate the association between groups with different eating habits, meal patterns, breakfast patterns, gender, age, and language spoken at home. Parental education level was not included in this analysis as it was only available for 5572 children. All statistical analyses were conducted using SPSS statistical software version 24.0 and we adopted a 5% statistical significance level for all tests.

## Results

There were 5564 (52.6%) girls and 5005 (47.4%) boys included in the analysis. Average age was 11.14 (± 0.85) years, in which 61.1% (*n* = 6457) of participants were 11 years old. Among children with information available on parental education level (*n* = 5572; 52.7%), 55% (*n* = 3063) had parents with a high education level and 45% (*n* = 2509) had parents with low education level. Regular meal pattern was observed in 75.7% (*n* = 8001) of children, meaning that they had school lunch and dinner every school day, and regular breakfast pattern was observed in 81.0% (*n* = 8563).

**Fig. 1:**
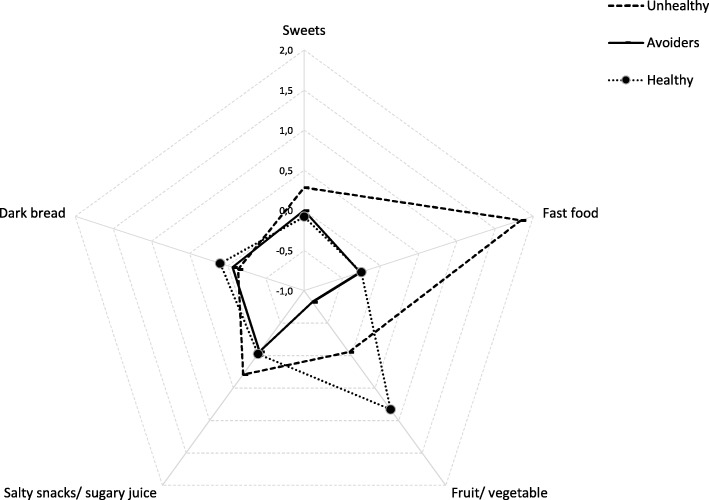
Average factor loads obtained from factor analysis for unhealthy eaters (unhealthy), fruit and vegetable avoiders (avoiders), and healthy eaters (healthy)

Factor analysis revealed five factors with high adaptability to the original data (KMO = 0.778; *p* < 0.001 for Bartlett’s sphericity test) and a high explanation of the variability of the data (70.1%) (Table 1). These factors represented five food groups: fast food (pizza; hamburger or hot dog); sweets (biscuits/cookies; sweet pastry; ice cream), salty snacks and sugary juice drinks; dark grain bread; and fruits and vegetables (fruits or berries; fresh or grated vegetables). From these factors, we obtained three groups with different eating habits: unhealthy eaters (12.3%; *n* = 1298), fruit and vegetables avoiders (43.3%; *n* = 4610), and healthy eaters (44.1%; *n* = 4661) (factor loads can be seen in Fig. 1).

**Table 1:** Factor loads for each food items used in factor analysis and percentage of variance explained by each factor

	Factors				
	Sweets	Fast food	Fruit/ vegetable	Salty snacks/ sugary juice	Dark grain bread
Pizza	0.156	0.809	−0.013	0.026	0.040
Hamburger or hot dog	0.123	0.765	−0.076	0.181	−0.046
Biscuits/cookies	0.767	0.094	−0.055	0.237	0.125
Sweet pastry	0.741	0.156	−0.039	0.206	0.114
Ice cream	0.652	0.326	0.176	−0.095	− 0.423
Salty snacks	0.219	0.466	−0.041	0.517	−0.037
Sugary juice drink (squash)	0.212	0.097	0.091	0.856	−0.068
Dark grain bread	0.134	0.026	0.310	−0.105	0.847
Fruit or berries	0.034	0.003	0.841	0.048	0.129
Fresh or grated vegetables/salad	−0.054	−0.093	0.842	0.015	0.101
Percentage of variance explained by each factor (total = 70%)	27.8%	17.7%	8.9%	8.3%	7.4%

To evaluate the association between all food items and eating habits, we categorized the 7-point scale into three categories (Table 2). This revealed that unhealthy eaters consumed more food items such as pizza, hamburger or hot dog, biscuits and cookies, sweet pastry, ice cream, salty snacks, sugary juice drinks, and soft drinks. Although fruit and vegetable avoiders ate less unhealthy food items, they consumed the least fruit or berries, fresh juice, and fresh or cooked vegetables. Healthy eaters were the most frequent consumers of dark grain bread, milk, fruits or berries, fresh juice, and fresh grated or cooked vegetables, and they ate less unhealthy foods.

**Table 2:** Consumption of the 14 food items included in the food frequency questionnaire among unhealthy eaters, fruit and vegetable avoiders, and healthy eaters

		Unhealthy eaters		Fruit and vegetable avoiders		Healthy eaters		Total		*p*-value^a^
		n	%	n	%	n	%	n	%	
Dark grain bread	Maximum once a week	323	24.9%	1358	29.5%	528	11.3%	2209	20.9%	< 0.001
	2–6 times per week	621	47.8%	2451	53.2%	2237	48.0%	5309	50.2%	
	At least once a day	354	27.3%	801	17.4%	1896	40.7%	3051	28.9%	
Fresh or grated vegetables/ salad	Maximum once a week	299	23.0%	1538	33.4%	33	0.7%	1870	17.7%	< 0.001
	2–6 times per week	639	49.2%	2682	58.2%	1144	24.5%	4465	42.2%	
	At least once a day	360	27.7%	390	8.5%	3484	74.7%	4234	40.1%	
Fruits or berries	Maximum once a week	251	19.3%	1992	43.2%	72	1.5%	2315	21.9%	< 0.001
	2–6 times per week	685	52.8%	2475	53.7%	1641	35.2%	4801	45.4%	
	At least once a day	362	27.9%	143	3.1%	2948	63.2%	3453	32.7%	
Sweet pastry	Less than once a week	493	38.0%	2896	62.8%	3115	66.8%	6504	61.5%	< 0.001
	Once a week	353	27.2%	1022	22.2%	1003	21.5%	2378	22.5%	
	more than once a week	452	34.8%	692	15.0%	543	11.6%	1687	16.0%	
Biscuits/ cookies	Less than once a week	352	27.1%	2023	43.9%	2294	49.2%	4669	44.2%	< 0.001
	Once a week	333	25.7%	1188	25.8%	1134	24.3%	2655	25.1%	
	more than once a week	613	47.2%	1399	30.3%	1233	26.5%	3245	30.7%	
Ice cream	Less than once a week	378	29.1%	3183	69.0%	2967	63.7%	6528	61.8%	< 0.001
	Once a week	359	27.7%	908	19.7%	957	20.5%	2224	21.0%	
	more than once a week	561	43.2%	519	11.3%	737	15.8%	1817	17.2%	
Sugary juice drinks	Less than once a week	296	22.8%	2229	48.4%	2026	43.5%	4551	43.1%	< 0.001
	Once a week	278	21.4%	1028	22.3%	1037	22.2%	2343	22.2%	
	more than once a week	724	55.8%	1353	29.3%	1598	34.3%	3675	34.8%	
Pizza	Not at all	21	1.6%	1150	24.9%	1182	25.4%	2353	22.3%	< 0.001
	Less than once a week	320	24.7%	3165	68.7%	3225	69.2%	6710	63.5%	
	At least once a week	957	73.7%	295	6.4%	254	5.4%	1506	14.2%	
Hamburger or hot dog	Not at all	29	2.2%	1255	27.2%	1573	33.7%	2857	27.0%	< 0.001
	Less than once a week	311	24.0%	3066	66.5%	2895	62.1%	6272	59.3%	
	At least once a week	958	73.8%	289	6.3%	193	4.1%	1440	13.6%	
Salty Snacks	Not at all	24	1.8%	448	9.7%	515	11.0%	987	9.3%	< 0.001
	Less than once a week	167	12.9%	2114	45.9%	2219	47.6%	4500	42.6%	
	At least once a week	1107	85.3%	2048	44.4%	1927	41.3%	5082	48.1%	
Milk or soured milk	Less than 4 times a week	230	17.7%	858	18.6%	458	9.8%	1546	14.6%	< 0.001
	Almost once a day	304	23.4%	1045	22.7%	689	14.8%	2038	19.3%	
	Several times a day	763	58.8%	2706	58.7%	3514	75.4%	6983	66.1%	
Cooked vegetables	Maximum once a week	775	59.7%	3273	71.0%	2066	44.3%	6114	57.9%	< 0.001
	Almost once a day	396	30.5%	1207	26.2%	1855	39.8%	3458	32.7%	
	Several times a day	127	9.8%	128	2.8%	738	15.8%	993	9.4%	
Fresh juice	Less than once a week	223	17.2%	1446	31.4%	888	19.1%	2557	24.2%	< 0.001
	1–4 times a week	533	41.1%	2082	45.2%	1700	36.5%	4315	40.8%	
	5–6 times a week or more	541	41.7%	1081	23.5%	2073	44.5%	3695	35.0%	
Soft drink	Less than once a week	249	19.2%	2296	49.8%	2536	54.4%	5081	48.1%	< 0.001
	Almost once a week	804	62.0%	2161	46.9%	2000	42.9%	4965	47.0%	
	5–6 times a week or more	244	18.8%	149	3.2%	123	2.6%	516	4.9%	

^a^results for Chi-square test

Unhealthy eaters showed the highest percentage of irregular meal patterns (31.8%; *n* = 413), and the highest percentage of irregular breakfast patterns (24.5%; *n* = 318). They were also the group with a high percentage of foreign children (4.9%; *n* = 64) and parents with low education level (55.0%; *n* = 343) compared with other groups (Table 3). Healthy eaters had a higher percentage of regular meal patterns (81.5%; *n* = 8001), regular breakfast patterns (86.3%; *n* = 4022), and had a higher percentage of parents with high education level (62.3%; *n* = 1567) (Table 3). Boys were over-represented among unhealthy eaters (61.5%; *n* = 798), as were girls among healthy eaters (59.5%; *n* = 2775) (Table 3). Irregular breakfast patterns were more common in girls (56.3%; *n* = 1130) than in boys. Moreover, there was a higher proportion of older children with irregular meal patterns (14.3%; *n* = 367) and irregular breakfast patterns (14.5%; *n* = 291) (Tables 4 and 5).

**Table 3:** Meal patterns, breakfast patterns, and socio-demographic characteristics of unhealthy eaters, fruit and vegetable avoiders, and healthy eaters

		Eating habits group						*p*-value^a^
		Unhealthy eaters		Fruit and vegetable avoiders		Healthy eaters		
		n	%	n	%	n	%	
Meal (lunch/ dinner) pattern	Irregular	413	31.8%	1294	28.1%	861	18.5%	< 0.001
	Regular	885	68.2%	3316	71.9%	3800	81.5%	
Breakfast pattern	Irregular	318	24.5%	1049	22.8%	639	13.7%	< 0.001
	Regular	980	75.5%	3561	77.2%	4022	86.3%	
Gender	Girl	500	38.5%	2289	49.7%	2775	59.5%	< 0.001
	Boy	798	61.5%	2321	50.3%	1886	40.5%	
Age	< 11 years	432	33.3%	1303	28.3%	1184	25.4%	< 0.001
	11 years	728	56.1%	2780	60.3%	2949	63.3%	
	> 11 years	138	10.6%	527	11.4%	528	11.3%	
Language spoken at home	Finnish	1182	91.1%	4324	93.8%	4344	93.2%	< 0.001
	Swedish	52	4.0%	185	4.0%	208	4.5%	
	Others	64	4.9%	101	2.2%	109	2.3%	
Parental education level^b^	Low	343	55.0%	1218	50.1%	948	37.7%	< 0.001
	High	281	45.0%	1215	49.9%	1567	62.3%	

^a^results for Chi-square test

^b^anything until technical high school was categorized as low education level; anything higher was classified as high education level

**Table 4:** Meal patterns (lunch and dinner) according to eating habits, breakfast patterns, and socio-demographic characteristics

		Meal pattern (lunch/dinner)				p-value^a^
		Irregular		Regular		
		n	%	n	%	
Eating habits	Unhealthy	413	16.1%	885	11.1%	< 0.001
	Avoider	1294	50.4%	3316	41.4%	
	Healthy	861	33.5%	3800	47.5%	
Breakfast pattern	Irregular	913	35.6%	1093	13.7%	< 0.001
	Regular	1655	64.4%	6908	86.3%	
Gender	Girls	1390	54.1%	4174	52.2%	0.084
	Boys	1178	45.9%	3827	47.8%	
Age	< 11 years	666	25.9%	2253	28.2%	< 0.001
	11 years	1535	59.8%	4922	61.5%	
	> 11 years	367	14.3%	826	10.3%	
Language spoken at home	Finnish	2362	92.0%	7488	93.6%	< 0.001
	Swedish	102	4.0%	343	4.3%	
	Others	104	4.0%	170	2.1%	
Parental education level^b^	Low	613	50.2%	1896	43.6%	< 0.001
	High	607	49.8%	2456	56.4%	

^a^results for Chi-square test

^b^anything until technical high school was categorized as low education level; anything higher was classified as high education level

**Table 5:** Breakfast patterns according to eating habits, meal patterns, and socio-demographic characteristics

		Breakfast pattern				*p*-value^a^
		Irregular		Regular		
		n	%	n	%	
Eating habits	Unhealthy	709	35.3%	3116	36.4%	< 0.001
	Avoider	674	33.6%	3066	35.8%	
	Healthy	623	31.1%	2381	27.8%	
Meal (lunch/ dinner) pattern	Irregular	913	45.5%	1655	19.3%	< 0.001
	Regular	1093	54.5%	6908	80.7%	
Gender	Girls	1130	56.3%	4434	51.8%	< 0.001
	Boys	876	43.7%	4129	48.2%	
Age	< 11 years	472	23.5%	2447	28.6%	< 0.001
	11 years	1243	62.0%	5214	60.9%	
	> 11 years	291	14.5%	902	10.5%	
Language spoken at home	Finnish	1962	91.9%	8174	93.3%	< 0.001
	Swedish	69	3.2%	404	4.6%	
	Others	105	4.9%	181	2.1%	
Parental education level ^b^	Low	571	57.7%	1999	42.6%	< 0.001
	High	419	42.3%	2697	57.4%	

^a^results for Chi-square test

^b^anything until technical high school was categorized as low education level; anything higher was classified as high education level

**Fig. 2:**
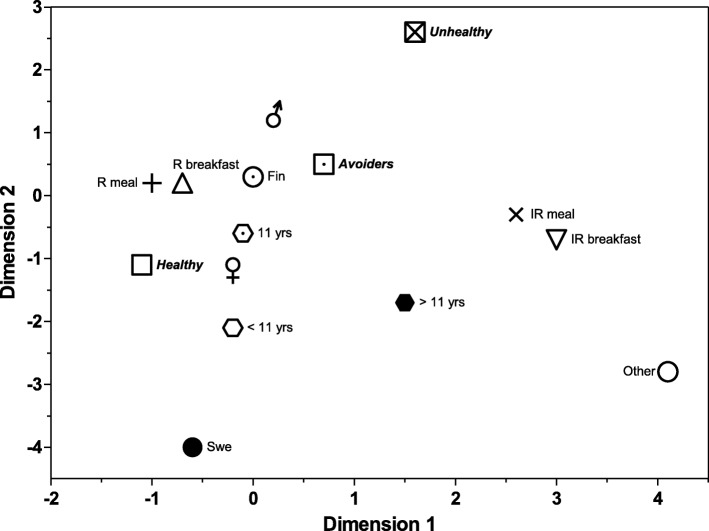
Map of results of the correspondence analysis. ^*^ eating habits are presented with different kind of squares, regular and irregular meal patterns with crosses, regular and irregular breakfast with triangles, languages with circles (Swe – Swedish; Fin – Finnish), age groups with hexagons and gender. R; regular, IR; irregular

The correspondence analysis summarized the associations of children’s characteristics with eating habits, meal patterns, and breakfast patterns, and confirmed the results presented in Tables 3-5. The resultant graphic representation of the combined results shows a clustering of irregular meal patterns, irregular breakfast patterns, foreign background, and older children (Fig. 2). Unhealthy eaters were more associated with male gender and younger age. Healthy eaters were clustered with regular meal pattern and regular breakfast pattern and were associated with female gender.

## Discussion

We identified three groups of children with different eating habits: unhealthy eaters, fruit and vegetable avoiders, and healthy eaters. The meal and breakfast patterns of these groups also differed, as did the socio-demographic characteristics: gender, age and language spoken at home.

All participants were pupils in elementary/primary public schools in Finland, where school lunch is served every school day, free of charge [34]. School lunch provides 20% of daily energy intake [9], underlining that most of the differences in adolescents’ food intake depend on food choices made outside school. Since we were interested in eating habits, we focused on key food items as indicators of healthy or unhealthy eating habits and also in those commonly consumed between meals or as snacks. Children may get snacks from vending machines, school kitchen or bring from home. In several schools in Finland, pupils are able to buy snacks from vending machines or from the school kitchen. Healthiness of these snacks are of concern, since it is difficult to monitor and even more difficult to intervene [9, 35].

We identified eating habits using factor analysis and cluster analysis. In total, five distinctive factors were identified: fast food, sweets, salty snacks and sugary juice drinks, dark grain bread, and fruits and vegetables. More generally, these five factors illustrated food items that were correlated with each other. Our results are somewhat similar to dietary patterns that have been described in the Finnish population [5, 12, 36, 37].

In our cohort, 34.7% of children consumed sugary juice drinks more than once a week, and 4.9% consumed soft drinks at least 5–6 times per week [11]. A sweet dietary pattern has been recognized in various previous nutrition studies in Finland [38, 39], including a study by Bingham et al., which noted that sweet foods constituted a notable part of the diet of Finnish army recruits, and were typically consumed as snacks between meals, or used to replace meals [39]. Moreover, sugar-sweetened drinks are common sources of sucrose in preschool and school-aged children [11]. Interestingly, 66.1% of our children consumed milk or sour milk several times a day, 32.4% reported eating cooked vegetables, most likely potatoes, almost once a day, and 29% had dark grain bread at least once a day, which illuminate the traditional Finnish dietary pattern [37]. Dark grain bread, especially rye bread is a traditional food item in Finland [37] and seems to be popular across different age groups, with similar patterns reported in young military recruits [39], young children [11], and pregnant women [5]. A dietary pattern with fruits and vegetables was identified in our children as well, with 32.7% reporting to eat fruits and berries and 40.1% reporting to eat fresh or grated vegetables at least once a day, which showed lower consumption frequency of vegetables, fruits and berries than recommended [40]. Our study did not provide information on quantity, only frequency of consumption. Previous studies support our findings and have reported similar or even lower portions for daily vegetable, fruit, and berry user among Finnish children and adolescents [9, 11, 12]. These foods are typically linked to healthy or health-conscience dietary patterns [5, 37, 38, 41], but less to the traditional Finnish diet [37].

In our study, 44.1% were healthy eaters and 43.6% were fruit and vegetable avoiders. The avoiders group ate less sweets and fast foods, but they did not choose fruits or vegetables either. Unhealthy eaters made up the smallest proportion of our cohort (12.3%). They mostly consumed fast food, sweets, and sugary drinks. Unhealthy eaters were mostly boys and younger children, and their parents had a lower education level compared with the other groups. The foods items characterizing the unhealthy eaters in our study were similar to those found in ISCOLE, a multi-national study [36]. Although it is unclear, the association between unhealthy diet and gender has been reported in several studies around the world, in which boys have consistently been over-represented in groups with unhealthy diet [42, 43]. It has been reported that girls eat more fruits and vegetables than boys [44]. This shows more healthy behaviour among girls, which is expected since it has been suggested that they also have a higher affinity for vegetables and have fewer perceived barriers to their consumption [44, 45]. A previous study pointed out that among male adolescents a healthier diet is associated with less peer pressure, and is positively correlated with adolescents’ self-confidence [46]. Thus, programs should work to change the perception of healthy eating so it is also seen as a masculine habit.

Fruit and vegetable avoiders ate the least fruits and vegetables, even lesser than unhealthy eaters. However, avoiders did not eat unhealthy foods, and the majority had both regular meal and regular breakfast consumption, suggesting they are less likely to eat or drink between meals. Nevertheless, several studies have shown that reduced consumption of fruits and vegetables is associated with overweight. One possible explanation for this group is a possible association between avoidance of fruit and vegetable consumption with overweight and under-reporting. Studies have shown under-reporting of food consumption is common in adolescents [47, 48]. The HELENA study reported that obese and overweight adolescents were more likely to under-report food intake, while underweight adolescents were more likely to over-report [47]. Older age is also associated with under-reporting of food and drink intakes among adolescents [48], and in our study a higher proportion of older children were observed both in the fruit and vegetable avoider and healthy eater groups.

Regular meal and breakfast consumption are part of the healthy diet [40], whereas unhealthy behaviours such as skipping breakfast or lunch or consuming high amounts of unhealthy food are associated with the development of non-communicable diseases, especially metabolic syndrome [4, 49, 50]. In this study, we evaluated meal patterns as lunch and dinner only, and looked at breakfast patterns separately. We found that most children consumed breakfast, as well as lunch and dinner every school day. In general, children with an irregular meal pattern had also an irregular breakfast pattern, which is considered unhealthy. Moreover, skipping lunch and breakfast increases the chance of an unhealthy diet among adults and adolescents in Nordic countries [19]. Much emphasis is placed on breakfast in school-aged children, as it is associated with the intake of nutrients that are important for young adolescents’ health [51, 52]. Skipping breakfast was more common in girls and older children, which is consistent with previous studies [30, 31, 51, 53]. The consumption of breakfast among women varies between countries, but a lower consumption has been noted among women from the Nordic countries [16]. However, in Finland, the consumption of daily breakfast in adolescents girls increased from 2002 to 2010, while this trend was reversed among boys [16].

We included language spoked at home and parental education level in order to evaluate the socioeconomic status of children. Previous Finnish studies have shown that Swedish-speakers have higher socioeconomic status, while immigrants have lower income than the general Finnish population [23, 24, 54]. The association between low socioeconomic status and unhealthy diet behaviour is well-established [55–58]. In the present study, the association between parental education level and children’ eating habits and meal/breakfast patterns was only investigated in a subset of participants. Low parental education level and foreign language was more prominently associated with unhealthy eating habits, irregular meal patterns, and skipping breakfast. Our results are consistent with studies showing that lower socioeconomic status is associated with poor quality of the diet, high consumption of fast foods and sweets, and lower consumption of fruits and vegetables [56, 57]. The DIATROFI study showed that daily consumption of breakfast was associated with a higher socioeconomic status [57]. Students with low socioeconomic status have also been shown to have an increased risk of skipping breakfast, lunch, and dinner [58].

The large, nationwide cohort of children is an ultimate strength of our study. Although the participation rate was low (30%), the distribution of socio-demographic characteristics (such as BMI, gender, maternal language) in our cohort were similar to Finnish children population [20]. Moreover, this large sample size allowed us to identify three distinctive eating habits. Importantly, we were able to characterize a small group of children with unhealthy eating habits. Our findings are consistent with others previous studies, although the FFQ had a short number of food items, as usually is used in this type of school studies [18, 28, 29] since there are limitations in carrying out long questionnaires with children and adolescents [25, 26]. We lacked information on the whole diet e.g., food consumption during main meals and meal consumption during weekends. In addition, we were not able to calculate total energy intake since FFQ included only a limited number of food items. Moreover, the FFQ has not been validated. We could assume some inaccuracy in food intake, since information was self-reported by 9–14-year-old children, but it was out of the scope of this study. However, an earlier study showed reliable results in similar FFQ among 11-year old and older children [29]. Furthermore, a qualitative evaluation of the questionnaire in this age group was carried out at the beginning of the study to check the children’s cognitive maturity [20]. Another weakness was that parental education level was available for 57% of children, but despite this, our results were similar with those of other studies.

## Conclusion

In conclusion, we identified three groups of children with different eating habits: unhealthy eaters, fruit and vegetable avoiders, and healthy eaters. A low percentage of our children were unhealthy eaters, and a high proportion of these were boys. In addition, association between unhealthy eating habits and irregular meal/breakfast patterns were observed. Special attention should be paid to avoider eaters since they have a low consumption of fruits, berries and vegetables, which is typically associated with increased risk of obesity and common chronic diseases. Most of the children had regular meal and regular breakfast patterns. In general, those with irregular meal patterns tended to have irregular breakfast patterns as well, although skipping breakfast was more common among girls and older children. This is the first study to evaluate eating habits and their association with meal patterns and breakfast consumption among young children in Finland. Our results increase understanding on unhealthy eating habits in children and provide further arguments for public health interventions in order to improve healthy eating behaviours.

## Abbrevations

FFQ: Food frequency questionnaire.
Fin-HIT: The Finnish Health in Teens.
HBSC: Health Behaviour in School-Aged Children.
KMO: The Kaiser-Meyer-Olklin.
